# Self-organization in a diversity induced thermodynamics

**DOI:** 10.1371/journal.pone.0188753

**Published:** 2017-12-08

**Authors:** Alessandro Scirè, Valerio Annovazzi-Lodi

**Affiliations:** Dipartimento di Ingegneria Industriale e dell’Informazione, Università di Pavia, Via Ferrata 5, I-27100, Pavia, Italy; Lanzhou University of Technology, CHINA

## Abstract

In this work we show how global self-organized patterns can come out of a disordered ensemble of point oscillators, as a result of a deterministic, and not of a random, cooperative process. The resulting system dynamics has many characteristics of classical thermodynamics. To this end, a modified Kuramoto model is introduced, by including Euclidean degrees of freedom and particle polarity. The standard deviation of the frequency distribution is the disorder parameter, *diversity*, acting as temperature, which is both a source of motion and of disorder. For zero and low diversity, robust static phase-synchronized patterns (crystals) appear, and the problem reverts to a generic dissipative many-body problem. From small to moderate diversity crystals display vibrations followed by structure disintegration in a competition of smaller dynamic patterns, internally synchronized, each of which is capable to manage its internal diversity. In this process a huge variety of self-organized dynamic shapes is formed. Such patterns can be seen again as (more complex) oscillators, where the same description can be applied in turn, renormalizing the problem to a bigger scale, opening the possibility of pattern evolution. The interaction functions are kept local because our idea is to build a system able to produce global patterns when its constituents only interact at the bond scale. By further increasing the oscillator diversity, the dynamics becomes erratic, dynamic patterns show short lifetime, and finally disappear for high diversity. Results are neither qualitatively dependent on the specific choice of the interaction functions nor on the shape of the probability function assumed for the frequencies. The system shows a phase transition and a critical behaviour for a specific value of diversity.

## Introduction

Whereas atomic and sub-atomic physics is moved by the quest of the fundamental building block, chemistry and biology have collected and studied many cases of self-organization [1], [2]. Self-organization is a process in which a global pattern emerges from interactions among lower-level components of a system by solely means of local information, without reference to the global pattern. Such process is observed in many different circumstances, ranging from systems under thermodynamic control (spontaneous processes with a negative free-energy change), such as supramolecular complexes [3], crystallization [4], surfactant aggregation [5], certain nano-structures [6], to protein folding [7], protein assembly [8], and DNA duplexing [9], as well as in systems under kinetic control (biological systems with genomic, enzymatic and/or evolutionary control), such as virus assembly [10], formation of beehive and anthill [11], tissue formation [12], swarm intelligence [13]. Out-of-equilibrium systems (non-linear dynamic processes), such as the Zhabotinsky-Belousov reaction [14] and other oscillating reactions, as well as convection phenomena [15] show self-organization of the pattern formation. Other examples of self-organization phenomena have been investigated, such as biological rithms [16], pattern formation and collective behaviour of neurons and in neuron networks [17], [18], [19], [20], patterns in excitable media [21], [22], [23], [24], quantum gravity [25], mobile networks [26]. Social systems also stem from self-organizing processes [27], e.g. human enterprises that form out of self-imposed rules, such as business companies, political parties, families, tribes, and spontaneous forms of collective arts as theater or dance.

The above examples, in a global view, show that self-organization is a ubiquitous and interdisciplinary process, and address the general question about how the self-assembly (auto-catalysis) of ordered structures, with growing complexity and emergent properties, takes place. If on one side noise and fluctuations were found to have a constructive role respect to pattern formation [28], [29], [30], on the other side diversity was recently found to be able to produce coherent collective pulsations out of a disordered ensemble of coupled oscillators [31], [32]. Diversity indeed appears to be a crucial ingredient for self-organization and the reason is that, if the elements are all equal to each other, there is no basis to self-organize, because no flux of information is necessary, and no criteria exists for a choice [33]. Whereas many unexplained collective behaviors—e.g. self-assembled chirality [34]—seem to stem from a transfer of information to the bond-length scale of size, many social patterns stem from relational choices based on affinity [35].

In this work, we explore the possibility of building a thermodynamics based on diversity, with emphasis on the self-organization properties and pattern formation. To do that we take profit of a theory that set a link between diversity and phase transitions, i.e., the Kuramoto model.

The Kuramoto model [36], first proposed by Yoshiki Kuramoto, is a mathematical model used to describe synchronization in a large set of coupled oscillators. It was found representative for the behavior of chemical and biological oscillators [37], and it has found widespread applications, e.g., in neuroscience or oscillating flame dynamics [38]. Kuramoto model set a link between collective synchronization and phase transitions. Indeed, the Kuramoto order parameter vs. oscillators natural frequency diversity shows a behavior parallel to spin magnetization in ferromagnetic media vs. temperature. Oscillators diversity acts as the temperature, for being a source of disorder.

In this work we consider a mechanics of interacting material points in a Euclidean space, each given an additional degree of freedom, a phase. Each phase-point is driven by an internal frequency which is the source of motion and a local parameter. Assuming a statistical distribution of such frequencies, a source of disorder is included, when many points are considered. Moreover, as a further element of novelty respect to Kuramoto, we include *polarity* in the model. The reason is that polarity is a natural characteristic of the large majority of observable systems, from physical to chemical to biological and social systems. Polarity is an intrinsic geometric richness, that allows the unfolding of complex structures, by alternating complementary elements. For low diversity in the local frequencies, oscillator phases synchronize, and a static global pattern (crystal) of synchronized points is the global attractor. By increasing oscillators diversity, desynchronization occurs, parallel to Kuramoto theory. Such transition takes place as follows: the crystal starts to vibrate, developing self-organized internal pulsations that lead to structure disintegration in a competition of dynamic smaller patterns, which are internally synchronized and show robustness and adaptability. By further increasing the oscillators diversity, the patterns finally disappear. Results are neither qualitatively dependent on the specific choice of the interaction functions nor on the shape of the probability function selected for the frequencies. The interaction function is kept local because our idea is to build a system able to produce global patterns when its constituents interact at the bond scale. Such global patterns can be regarded as self-organized structures. The system shows a phase transition and a critical behavior for a specific value of diversity.

## Results

### The model

Each point *i* = 1, …*N* is described by a position vector **x**_*i*_ embedded in an Euclidean space, a phase *ϕ*_*i*_ embedded in a circle *S*^1^, as dynamic variables, and by a frequency *ω*_*i*_ as local parameter. The proposed model reads

$${\dot{x}}_{i}=\overset{N}{\underset{j=1}{\sum }}f_{ij}\left(x_{i},x_{j}\right)\;\text{cos}(\phi_{j}-\phi_{i}),$$ (1)

$${\dot{\phi }}_{i}=\omega_{i}+\overset{N}{\underset{j=1}{\sum }}\gamma_{ij}g_{ij}\left(x_{i},x_{j}\right)\;\text{sin}(\phi_{j}-\phi_{i}),$$ (2)

and since we want particles to interact at the bond scale, we choose local interactions, and one possible choice is

$$\begin{array}{c} f_{ij}=r_{ij}e^{-r_{ij}^{2}}, \end{array}$$ (3)

$$\begin{array}{c} g_{ij}=e^{-r_{ij}^{2}}, \end{array}$$ (4)

$$\begin{array}{c} r_{ij}=x_{i}-x_{j}, \end{array}$$ (5)

$$\begin{array}{c} r_{ij}=|x_{i}-x_{j}|, \end{array}$$ (6)

where the exponential decay defines a characteristic interaction length *L* = 1. The coefficients *γ*_*ij*_ = ±1 express the particle polarity. In this context polarity means that two different kinds of particles are considered: when particles of the same kind are interacting, the Kuramoto force in Eq (2) is repulsive (i.e. *γ*_*ij*_ = 1 thus *ϕ*_*i*_ and *ϕ*_*j*_ are pushed to synchronize in phase), while when particles of different kind are interacting, the Kuramoto force in Eq (2) is attractive (i.e. *γ*_*ij*_ = −1 thus *ϕ*_*i*_ and *ϕ*_*j*_ are pulled to synchronize out of phase). Polarity is a characteristic that emerges when two or more particles interact. It is a local property, but it shows up in the interaction with other elements. We refer to these types of particles as “circles” or “squares”. This is similar to the electric charge, since one cannot say if an isolated particle has a positive or a negative charge. In the following we will show that, when no disorder is included, the effect of polarity is indeed the same as for the electric charge in Newton’s dynamics, i.e, it determines the sign of the interaction forces in Eq (1), making them attractive or repulsive.

### Two particles

The simplest case is that of two particles in one spatial dimension. Eqs (1) and (2) yield

$$\begin{array}{c} \dot{x}=xe^{-x^{2}}\cos\left(\phi \right), \end{array}$$ (7)

$$\begin{array}{c} \dot{\phi }=\Delta -\gamma e^{-x^{2}}\sin\left(\phi \right), \end{array}$$ (8)

where

$$\begin{array}{c} x=x_{2}-x_{1} \end{array}$$ (9)

$$\begin{array}{c} \phi =\phi_{2}-\phi_{1} \end{array}$$ (10)

$$\begin{array}{c} \Delta =\omega_{2}-\omega_{1}, \end{array}$$ (11)

are the relative variables, and *γ* = −1 or *γ* = 1 if a circle and a square or two squares (two circles) are considered, respectively. For small relative displacement *x* ≪ 1 Eqs (7) and (8) decouple and the phase Eq (8) takes the form of the well known Adler equation [39]

$$\begin{array}{c} \dot{\phi }=\Delta -\gamma \sin\left(\phi \right). \end{array}$$ (12)

**Fig 1:**
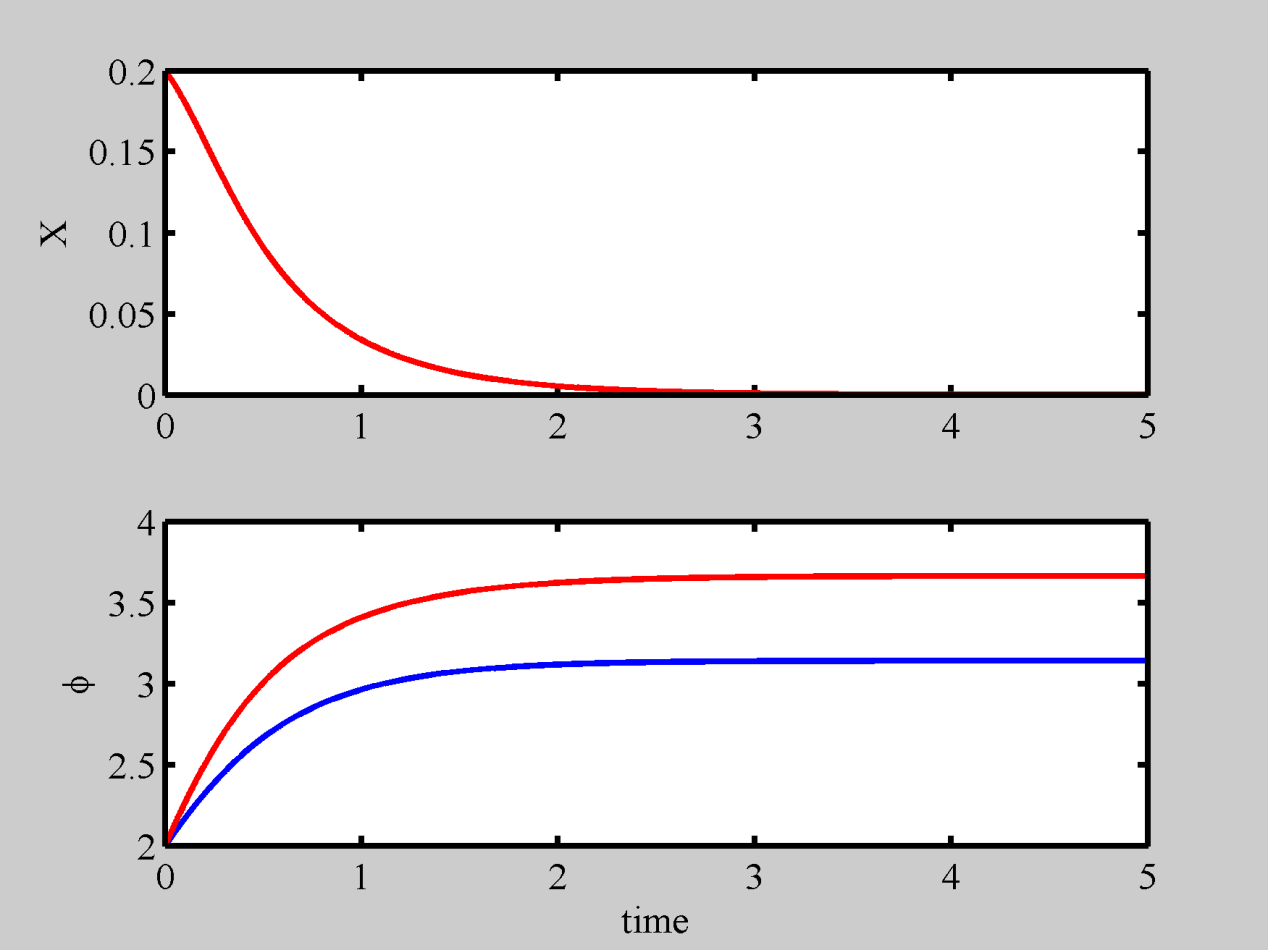
Two particles synchronization dynamics. Relative position dynamics (upper panel) and relative phase dynamics (lower panel) for a two particle system with no diversity (blue) and low diversity (Δ = 0.5, red) and different polarity (*γ* = −1). Particles glue together in an out-of-phase synchronization. Since identical starting conditions have been selected (x = 0.2, *ϕ* = 2), in the upper panel the curves are perfectly superposed.

For small diversity in the frequency difference (Δ < 1) the phases of the two particles synchronize, i.e. Eq 12 shows two static solutions *ϕ*_*in*_ = *arcsin*(Δ) and *ϕ*_*out*_ = *arcsin*(Δ) + *π*, that we call *in-phase* and *out-of-phase* solutions, respectively. If *γ* = −1 the fixed point *x* = 0, *ϕ*_*out*_ results to be a stable solution, so the two particles glue together *out-of-phase* (by *π*, for Δ = 0), as shown in Fig 1; if *γ* = 1 the (limit) fixed point *x* → ∞, *ϕ*_*in*_ results to be a stable solution, so the two particles repell each other *in-phase*. It is interesting to observe that the dynamics does not significantly change for low, non-zero, diversity (Δ < 1). This can be appreciated from Fig 1, where we have selected the same starting conditions for Δ = 0 and Δ = 0.5, getting identical evolution for position, and similar evolution for phase.

**Fig 2:**
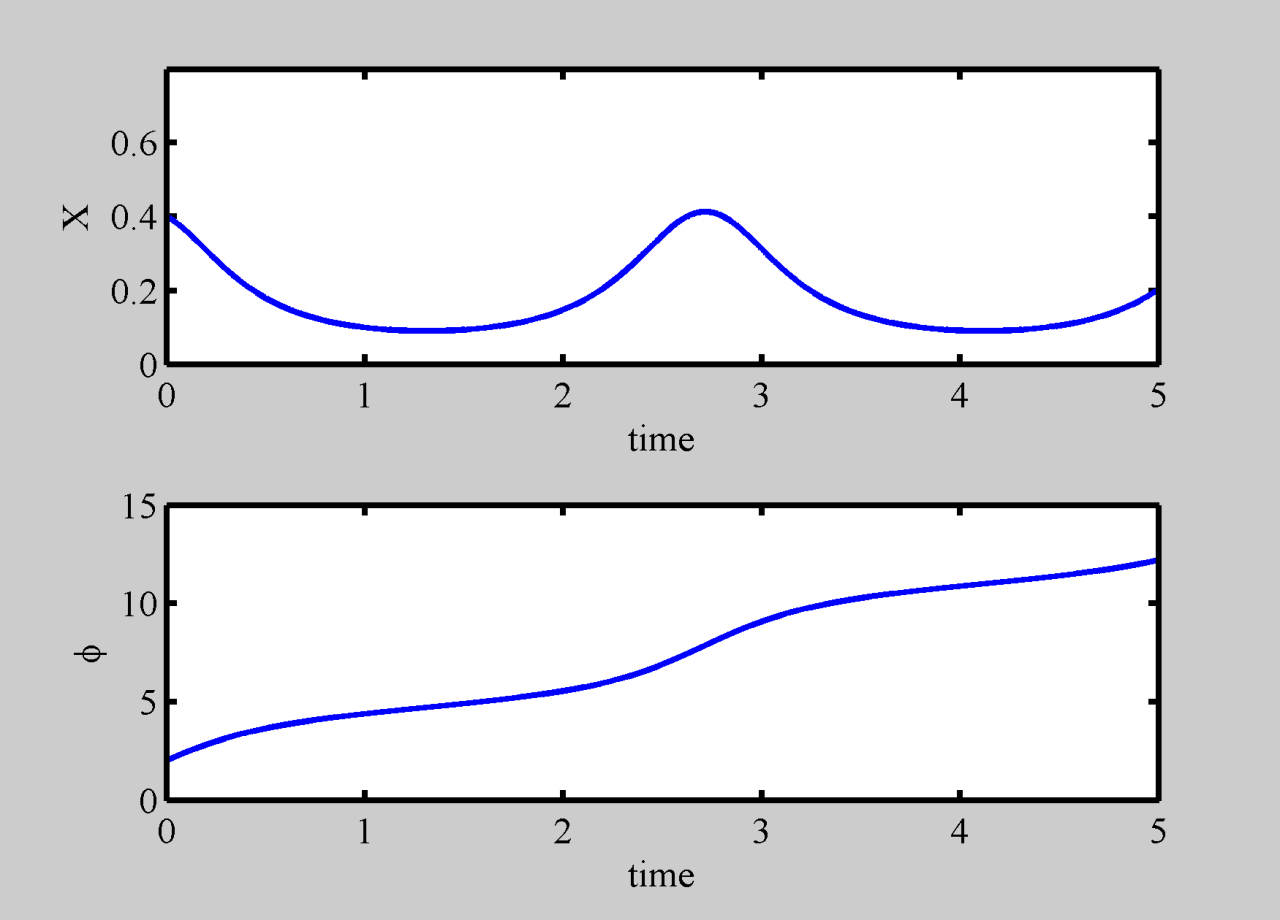
Two particle desynchronization dynamics. Relative position dynamics (upper panel) and relative phase dynamics (lower panel) for a two particle system with high diversity (Δ = 3). Particles oscillate while the relative phase is running (initial conditions: x = 0.4, *ϕ* = 4).

When Δ > 1 phase desynchronization occurs and the fixed points disappear via saddle node bifurcation, leading to oscillations in the relative position *x* and relative phase running, as shown in Fig 2. We have implemented other (polynomial) types of interaction decay dependences on distance, obtaining the same scenario.

In conclusion, in the case of two particles, we found them glue together (a molecule) for different polarity, while they repel and separate for identical polarity. The particles of the molecule oscillate with respect to each other as diversity increases. The results shown in Figs 1 and 2 do not depend on initial conditions, even though the transient may be different.

For a larger number of particles the complexity of the problem quickly grows.

### Many body problem. A diversity induced thermodynamics

Considering many particles including a statistical distribution of the local frequencies *ω*_*i*_, the system exhibits many properties of classical thermodynamics. Local frequencies are both a source of motion and of disorder, parallel to noise terms in Langevin formulation. If all frequencies have the same value (that can be set to zero, i.e. *ω*_*i*_ = 0 ∀*i*) the system drops into the synchronization manifold, which represents the “zero temperature” configuration, when no diversity is included in the system. In the synchronization manifold the effect of polarity (i.e. the coefficients *γ*_*ij*_) is the same as the electric charge in Newton’s dynamics, i.e. it determines the sign of the forces in Eq (1), making them attractive or repulsive. Indeed, *ϕ*_*i*_ − *ϕ*_*j*_ = 0 if *γ*_*ij*_ = 1 while *ϕ*_*i*_ − *ϕ*_*j*_ = *π* if *γ*_*ij*_ = −1, and Eqs (1) and (2) can be written as

$$\begin{array}{c} {\dot{x}}_{i}=\sum_{j=1}^{N}\gamma_{ij}f_{ij}, \end{array}$$ (13)

i.e., a generic many body dissipative mechanical problem where some interactions are attractive and some repulsive. If *diversity* in the local frequencies is included, phases start to desynchronize and the rotating terms *cos*(*ϕ*_*j*_ − *ϕ*_*i*_) in the space equation alter the interaction forces ***f***_*ij*_ producing a loosening in the spatial bondings and a subsequent phase transition, that mimics the thermodynamic transition from solid to liquid, to gas. In the following we assume a zero mean Gaussian distribution for *ω*_*i*_ with standard deviation *σ*, which acts as the system “temperature”.

**Fig 3:**
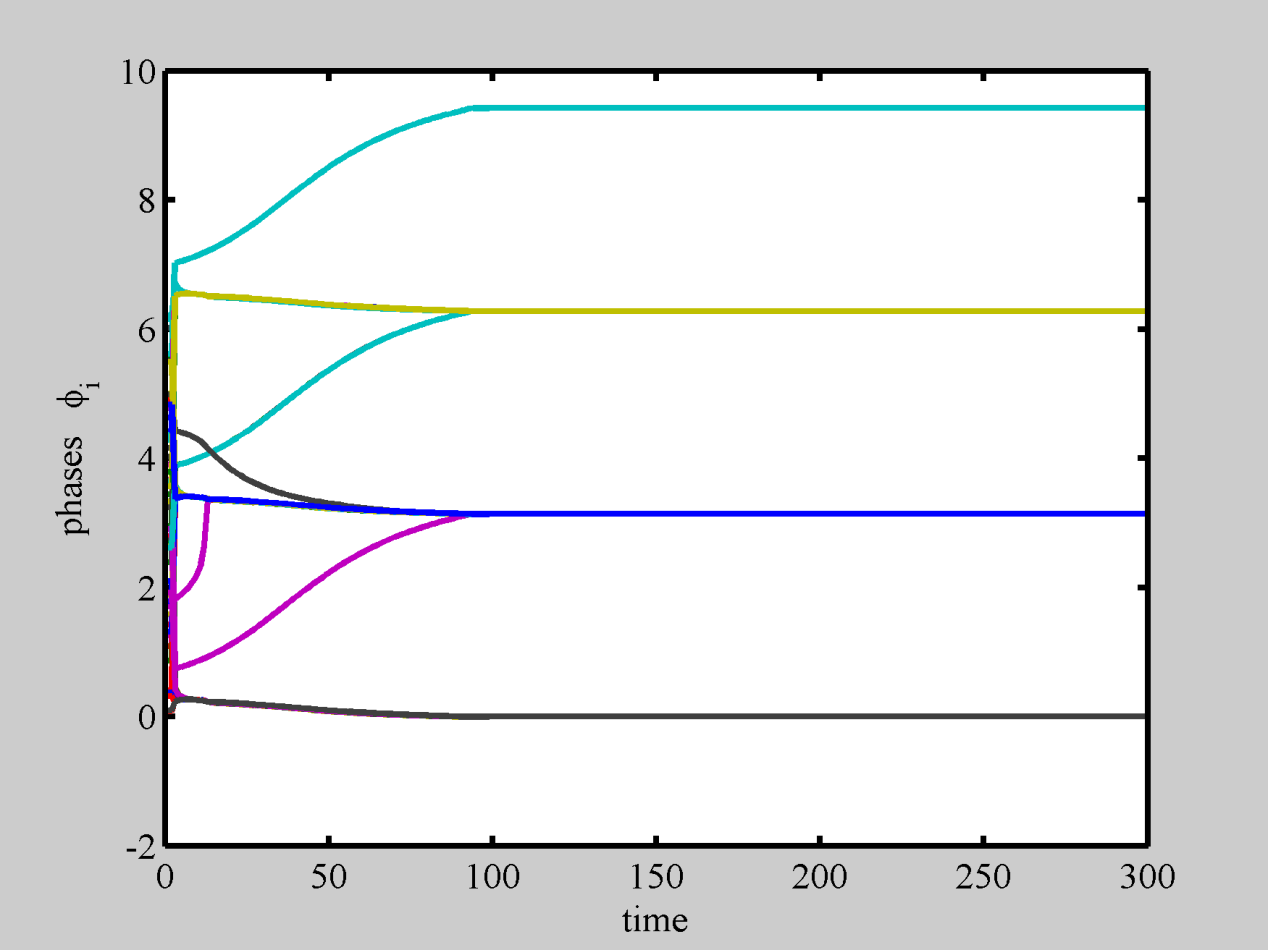
Even crystal formation. Phase dynamics. Even crystal formation, starting from random initial conditions spread in the plane (***x***_*j*_(0) > 1 ∀*j*), with no diversity (*σ* = 0). After a transient in which odd and even crystal compete, phases reach their static values. Different color represent different particles, only five colors have been used. *N* = 50.

**Fig 4:**
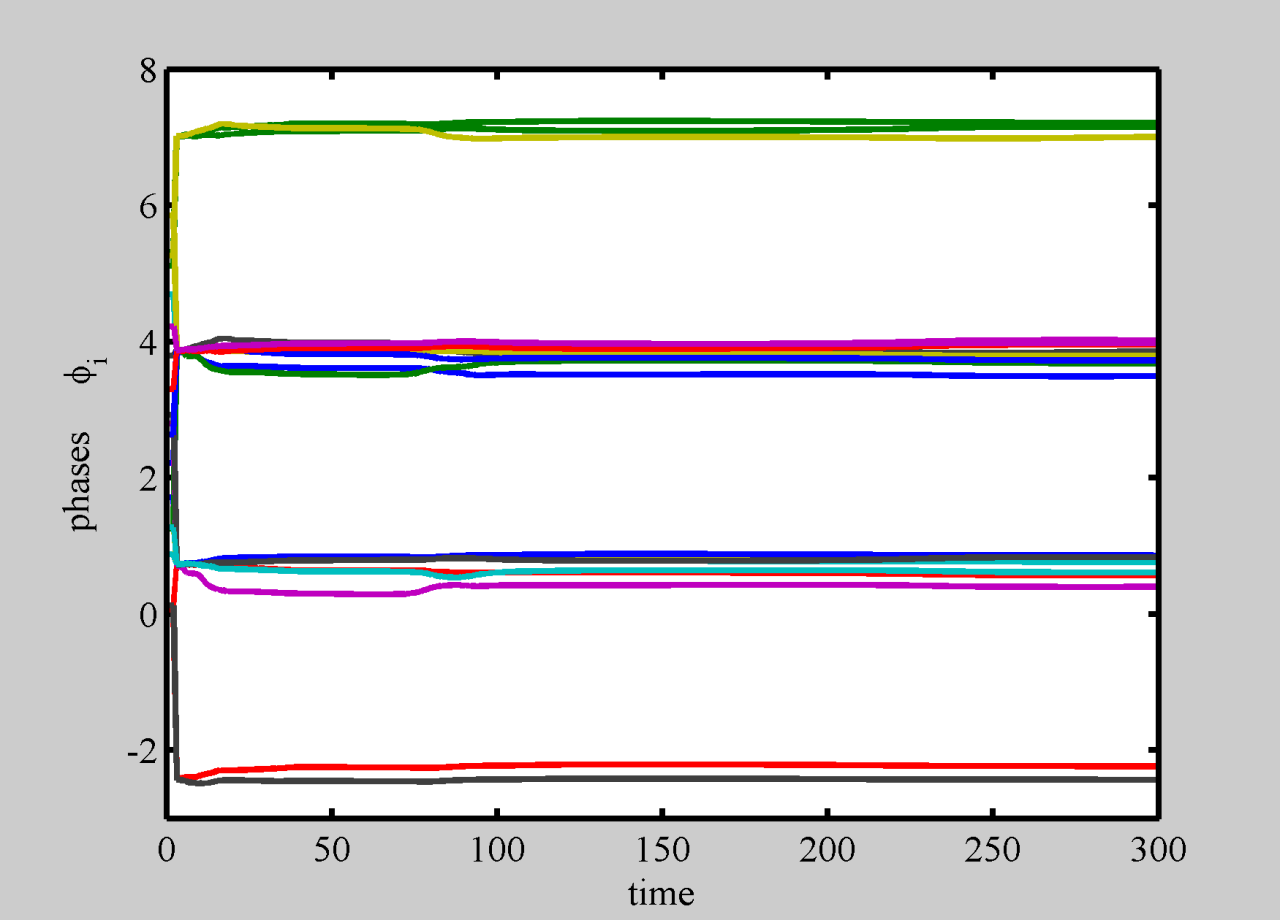
Phase dynamics for small diversity. Odd crystal dynamics for non zero, but low, diversity (*σ* = 0.25). Phases start to oscillate, synchronization is deteriorated (*N* = 21).

**Fig 5:**
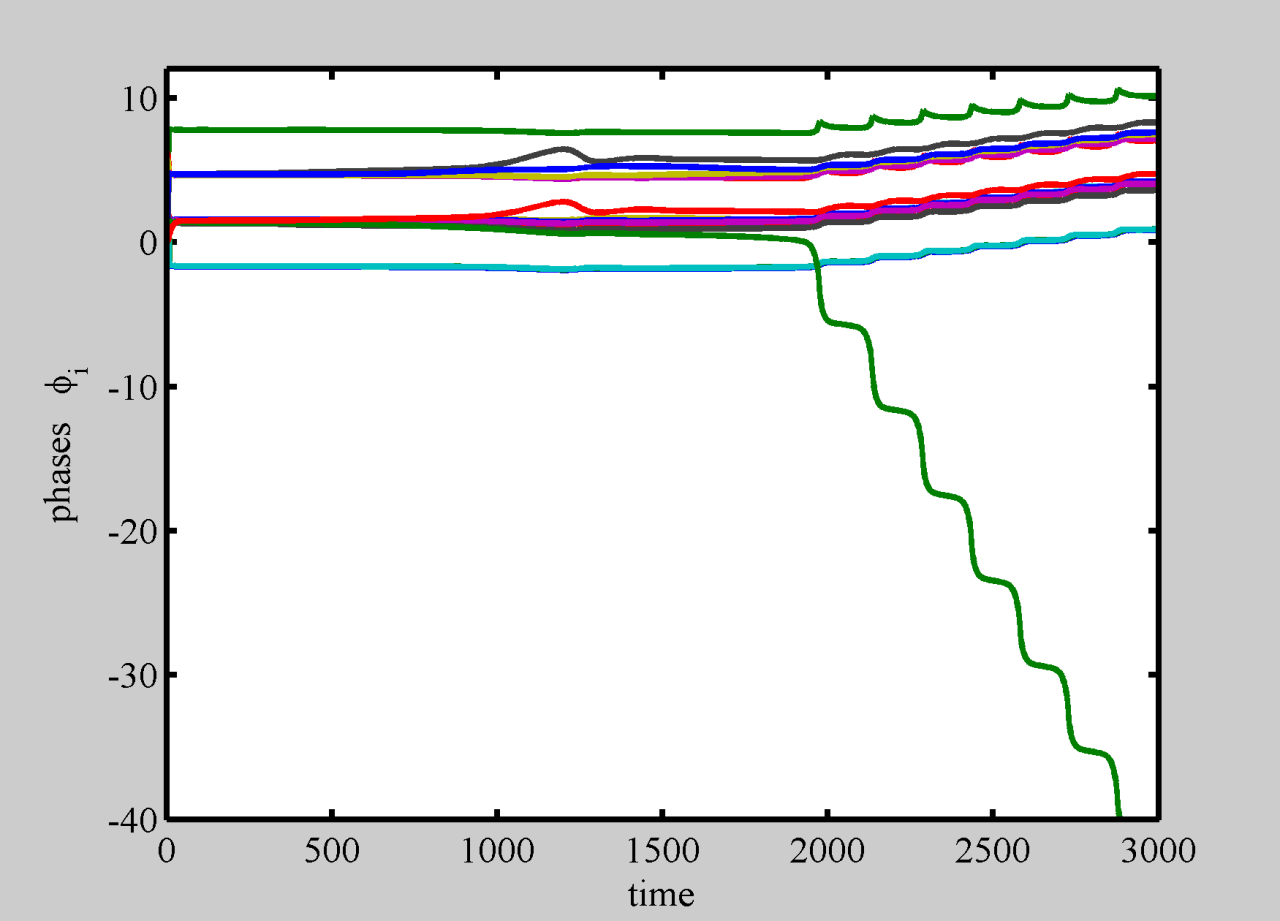
Example of phase dynamics in diversity induced collective pulsations. Evolution of phase vs. time for moderate diversity, with phase unlocking of one oscillator (*N* = 17 and *σ* = 0.4).

**Fig 6:**
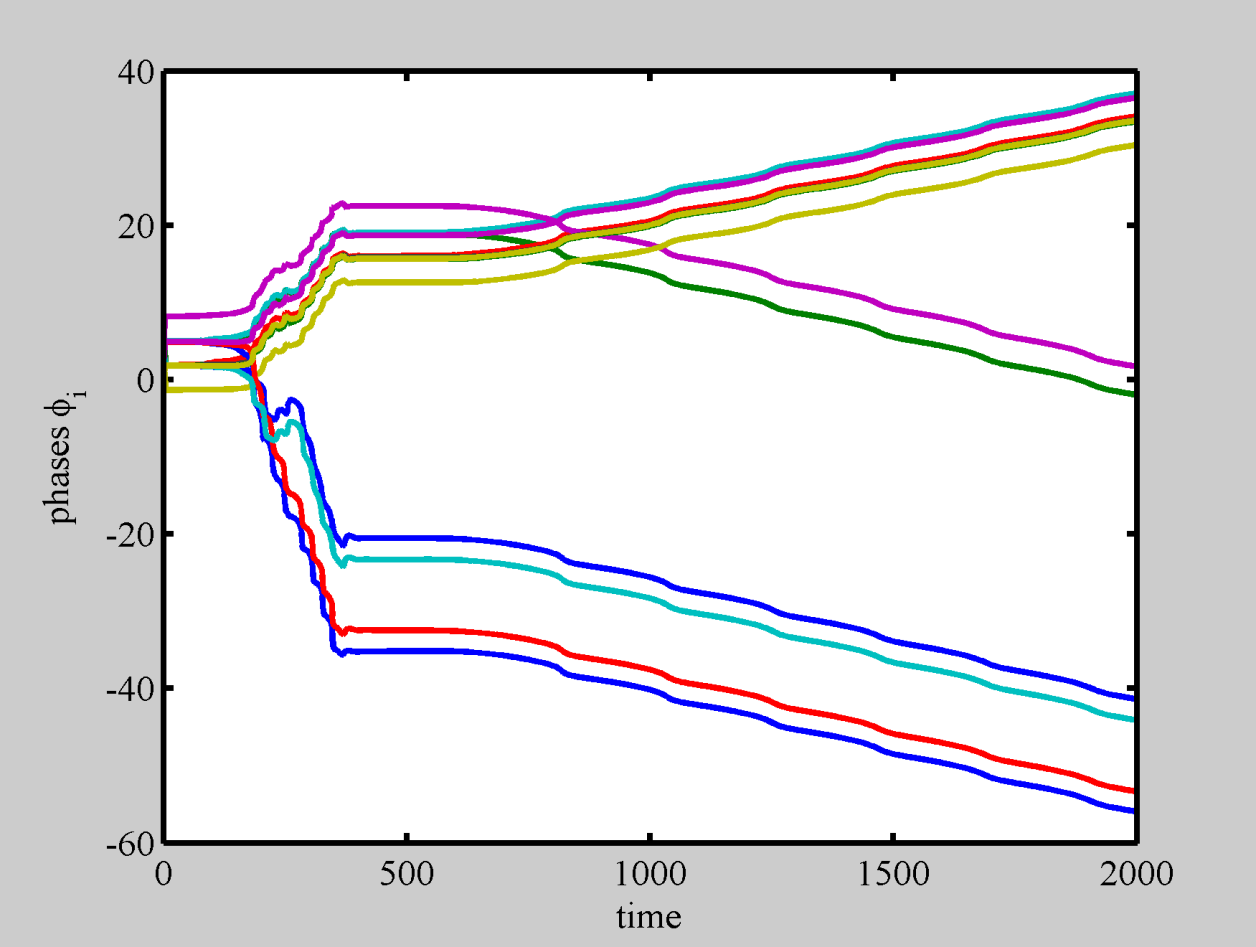
Phase dynamics for pattern breaking. For larger diversity, a single pattern is no longer sustainable, and diversity induced pulsations lead to pattern separation in two—internally synchronized—populations. (*N* = 13 and *σ* = 0.5).

**Fig 7:**
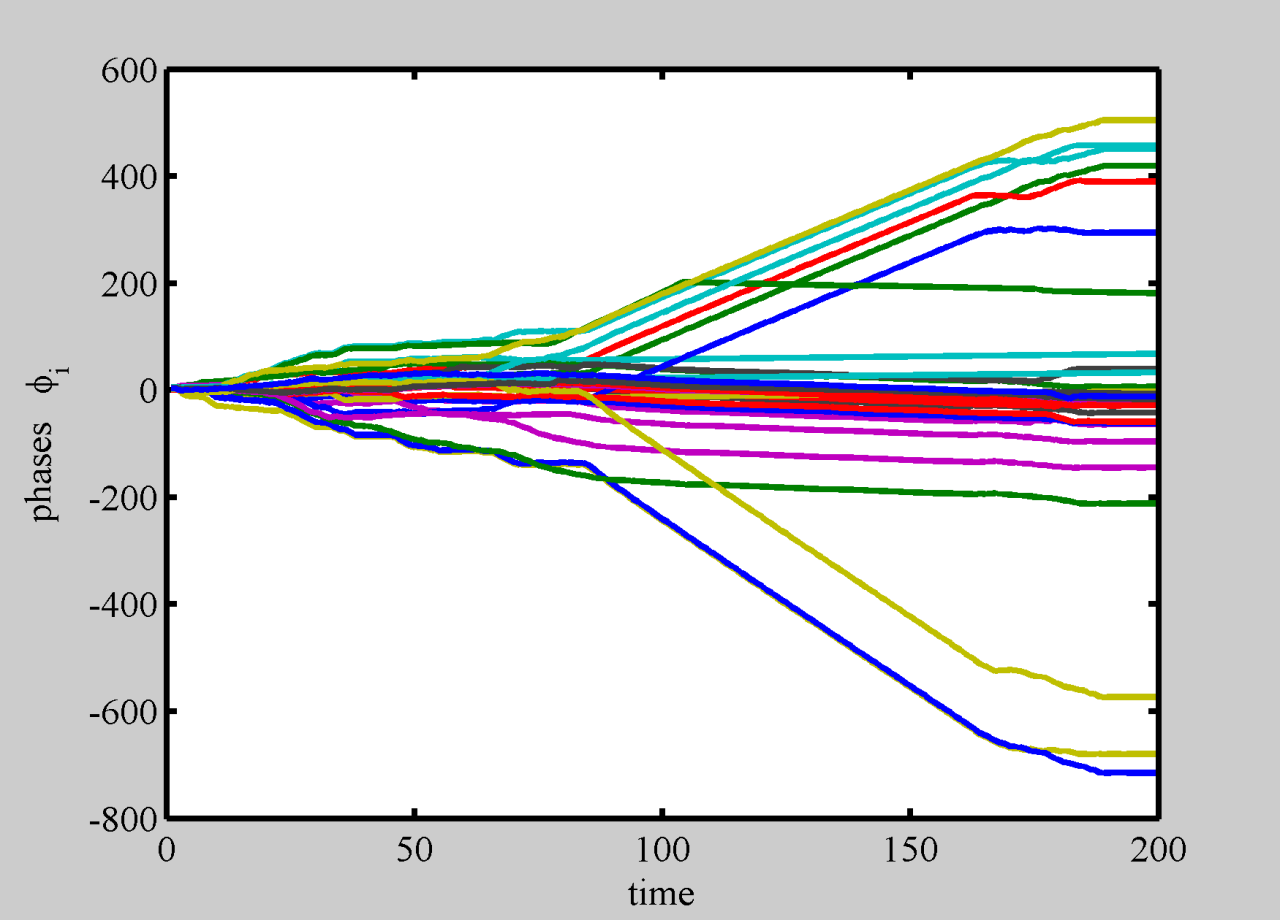
Phase dynamics for multiple patterns competition. Still larger diversity (i.e., still higher “temperature”), causes most phases to desynchronize. (*N* = 29 and *σ* = 0.7).

**Fig 8:**
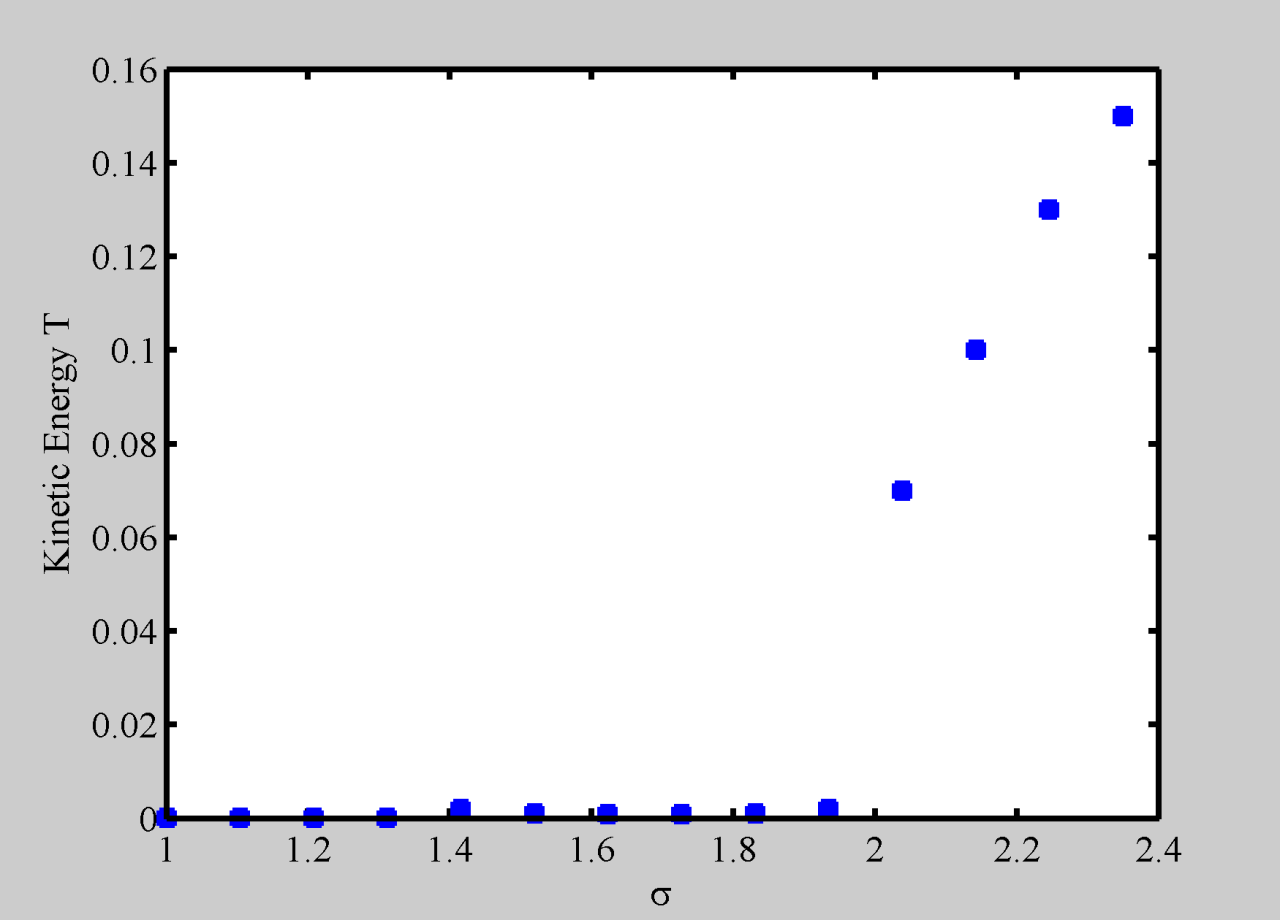
Average kinetic energy versus diversity. Critical behaviour of kinetic energy of the system vs. *σ*, showing a phase transition. *N* = 100.

**Fig 9:**
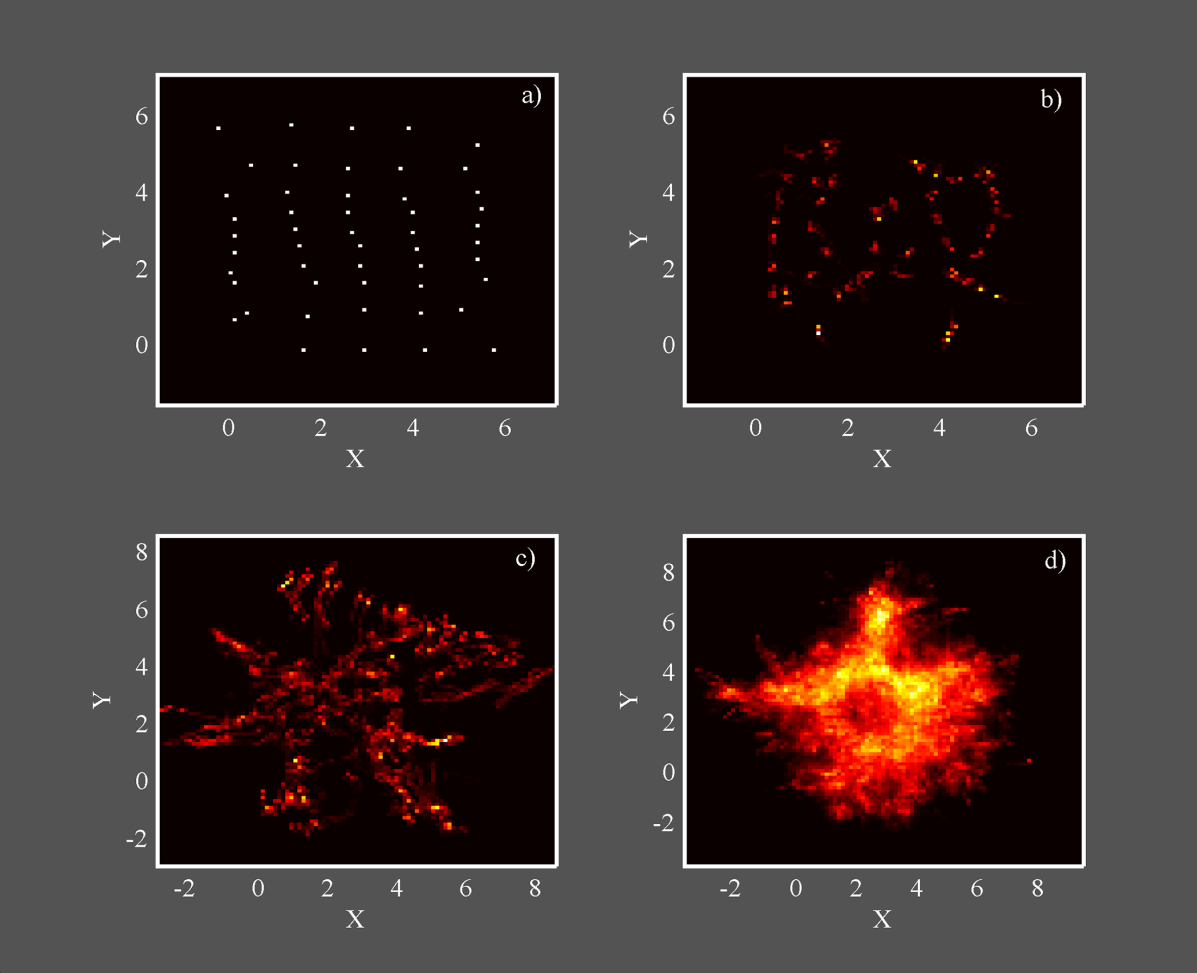
System evolution for N = 100. Occupation matrix for *N* = 100 and a) *σ* = 0, b) *σ* = 0.5, c) *σ* = 2, d) *σ* = 3, showing the complete system evolution for constant N and increasing diversity. The occupation matrix resolution is 100x100 pixels, and the total simulation time is *T* = 20000 for each panel. Clearer (darker) colors mean larger (smaller) occupation probability (increasing from black: 0, to white: 1, in a standard ‘hot’ color map).

We first consider the case in which *σ* = 0, i.e. identical oscillators, having the same local frequency, set to zero. In the following, we have assumed a neutral or quasi-neutral system (i.e, the number of circles and squares is equal, or differs only by 1), as it is reasonable to describe standard matter, and either spread or narrow intial conditions (i.e., large or small initial distances with respect to L = 1), corresponding to a relatively dilute or more condensed ensamble of particles. We have considered two spatial dimensions. Numerical simulations of Eqs (1) and (2) produce different solutions, depending on the number of particles N and on initial conditions, and remarkably including, for a large parameter set, the formation of static patterns, i.e. regular spatially extended structures (crystals), and a corresponding synchronization of local phases. Numerical simulations show the emergence of a competition between two kind of patterns, a first kind, we call *Even* (because it is the typical solution for even N), is made of glued couples of circles and squares, the second kind, we call *Odd* (because it is the typical solution for odd N), is made of strains of spatially separated alternating squares and circles. Both patterns show an out-of-phase synchronization, i.e., all squares have the same phase, all circles have the same phase, and between any square and circle there is a phase difference equal to *π*. To cover all relevant cases, in Figs 3 to 7, we show results for different values of N. In Figs 8 and 9, instead, results for the same N = 100 are shown.

In all figures we present in the following, and in movies, we have assumed uniformly distributed random initial conditions and Gaussian distributed oscillator frequencies. However, we have tested also uniform probability distribution for the natural frequencies, as well as polinomial instead of exponential interaction functions, finding the same scenarios. In figures, different colors represent different particles, with repetition, because only five colors have been used. In movies, instead, colors represent the phase evolution, i.e. same color means the same phase (mod 2*π*). In general, the actual shape of the obtained spatial patterns strongly depends on starting conditions. However, the general scenario does not change.

Movie S1 Video shows the numerical simulation of a population made of *N* = 50 particles, with wide initial distances (***x***_*j*_(0) > 1 ∀*j*), ending up in a static Even-crystal. Fig 3 shows phase dynamics vs. time for the same simulation.

Movie S2 Video shows the self-organization of a system made by *N* = 21 particles, where we have assumed (uniformly) random initial phases and small initial distances (***x***_*j*_(0) ≪ 1∀*j*) among the elements, so that all interact at *t* = 0. Phase dynamics (not shown) is very similar to the previous case. The result is a strainded Odd-crystal.

For non-zero, but small, *σ* (*σ* < 0.3) pattern vibrations take place, spatial structures deteriorate their regularity and phases start to oscillate. Now each particle is different so that the pattern has to adjust its structure, in order to accomodate each particle to a suitable place to sustain the collective structure. Movie S3 Video shows an example of such adaptability. The degree of diversity is still low, so the pattern can maintain a quasi-static integrity. Phase dynamics for this case is shown in Fig 4, exhibiting phase vibrations.

In order to investigate pattern robustness, we have implemented the following numerical experiment. After the pattern is formed, one element is moved away from the pattern, by increasing its position and phase of an arbitrary value. Movie S4 Video shows how the pattern (with no diversity) quickly reacts, by reincluding the perturbed element in a newly adapted structure. This characteristic persists in presence of small diversity.

Increasing diversity, the dynamic activity also increases and crystals loose stability. Many scenarios are possible. As a general trend, a single aggregation pattern is not sustainable if diversity becomes too large. Indeed, pattern disintegration takes place through the emergence of collective pulsations. Movie S5 Video shows an example of such collective pulsations, which are due to partial unlocking of one or more oscillators (Fig 5), which however remain spatially bounded, until the pattern eventually breaks. At this point, because of the complexity of the dynamics, the simple classification Even/Odd does not apply. We thus present selected numerical examples.

Indeed, we found that spontaneous internal oscillations lead to pattern separation into smaller structures, able to manage their internal diversity, and a huge variety of dynamically interacting shapes is formed. Movie S6 Video shows a realization in which a pattern spontaneously separates in two parts. Each part shows internal synchronization to the average frequency of its constituents. Phase dynamics for this situation (Fig 6) shows that the two patterns are made of synchronous elements. The so formed patterns are able to compete with other patterns in a complex dynamics, as shown in movie S7 Video and in Fig 7.

As *σ* further increases, progressively less organized dynamics take place. Elements move erratically, phases are desynchronized, the resulting patterns have short lifetimes, and finally no pattern emerges for very high *σ*. Still, the dynamics is deterministic.

It is worth noticing that, when synchronized patterns are formed, each of them can be seen, on its turn, as a (more complex) oscillator, able to synchronize again to affine structures, tolerating a certain amount of diversity, and so forth. A sort of Chinese box of synchronized shapes can form out spontaneously, opening the possibility of having *renormalized layers* of evolution, with increasing complexity. As a matter of fact, once patterns are formed they can be renormalized, i.e. considered as new points of a new point-like description on a larger scale, where they undergo the same process (synchronization) that previously created them, and so forth. Thus, starting from any arbitrary distributed and disordered point-like description, pattern formation gains growing complexity, moving up through evolutive layers. In this way a myriad of interacting dynamic shapes (*morphogenesis*) is spontaneously created by the cooperative synchronization process, especially where diversity is moderate, the equivalent of the “liquid” phase.

### Kinetic energy and phase transition

The existence of a phase transition between more and less organized patterns (crystal and liquid) can be confirmed by calculating the dynamic activity of the oscillators as a function of *σ*, which acts as the system temperature. Parallel to thermodynamics, we consider the kinetic energy of the system given by

$$\begin{array}{c} T=\frac{1}{N}\sum_{i=1}^{N}{<v_{i}}^{2}>, \end{array}$$ (14)

where $<v_{i}^{2}>$ are the time averaged particle square velocities and *N* the number of particles. For low diversity, the particles have fixed positions inside the crystal, their velocity is zero and the kinetic energy is also zero. A critical behavior appears at a special value of *σ* as shown in Fig 8, indicating the presence of a phase transition, where the crystal dissolves and particles are free to move. This transition is abrupt, as the melting of a real crystal. This finding is similar to what predicted by the Ising model [40], [41] describing the peculiar behavior of the specific heat of solids and of magnetization for low temperature.

Fig 8 suggests that, when considering a large number of oscillators, the global dynamics can be seen in terms of phase transitions, driven by the oscillators diversity. In the following, we better address this point and show that the complete evolution we have outlined in this paper can be found for constant N. In Fig 9 we have drawn the occupation matrix for *N* = 100 oscillators, with the same starting conditions, and four increasing values of diversity. In the occupation matrix, each element represents the probability of finding one oscillator in a small element *dxdy*, regardless it is a circle or square, along the full time dynamics. As starting conditions, we have chosen a regular 10x10 arrangement alternating circles and squares, separated by a distance equal to half the interaction length *L* (*L* = 1), with linearly increasing starting phases from *ϕ*_1_ = 0 in the left up corner, to *ϕ*_*N*_ = 2*π* in the right bottom corner. Panel a) shows the occupation matrix for zero diversity, where a static (even) crystal is formed. Panel b) shows the occupation matrix for *σ* = 0.5, i.e., on the left of the transition shown in Fig 8, and the result is a vibrating crystal with oscillators diffusion, and the structure still shows some regularity. Panel c) shows the occupation matrix for *σ* = 2, above the critical value, where a sort of “liquid” phase takes place, oscillators smoothly tend to occupy the interstitial spaces, and regularity is lost. Finally, panel d) shows the occupation matrix for high diversity *σ* = 3. The dynamics is now characterized by a high degree of disorder, resambling the motion of a gas.

## Conclusion

We have considered a mechanics of interacting material points, each given an additional degree of freedom, i.e. a phase. Each point is characterized by an internal frequency which is the source of motion. We have assumed a statistical distribution of those frequencies when many points are considered, and the standard deviation of such distribution as the global disorder parameter: diversity. For zero and low diversity, static patterns (crystals) appear. For moderate diversity the crystals start to vibrate, developing self-organized internal pulsations that lead to structure disintegration, progressively melting in a competition of dynamic patterns. When moderate diversity is included, a single static global pattern is no longer sustainable, and it breaks into smaller patterns, each of which is capable to manage its internal diversity. In this process a huge variety of self-organized dynamic shapes is formed. Moreover, when synchronized patterns are formed, each pattern can be seen again as a (more complex) oscillator able to synchronize in turn to similar structures, tolerating a certain amount of diversity, and so forth. From a conceptual point of view, the fact that self-organized patterns emerge as a result of a process, and not by mere chance, gives a significance to morphogenesis, because here the existence of a form means that an underlaying cooperative synchronization process is taking place.

Increasing further the oscillators diversity the dynamics ends being erratic and disorganized, dynamic patterns show short lifetime and finally disappear.

Results are neither qualitatively dependent on the specific choice of the interaction functions nor on the shape of the probability function chosen for the frequencies. The interaction functions are kept local because our idea is to build a system able to construct global patterns when its constituents interact at the bond scale. Such global patterns can be regarded as self-organized structures. The system shows a phase transition and a critical behavior for a specific value of diversity.

## Supporting information

S1 Video Even crystal formation with no diversity. Numerical simulations starting from (uniformly) random wide spread initial conditions for a population of *N* = 50 identical oscillators, *σ* = 0, with different polarity. Colors follow phase evolution: particles with the same color have the same phase value in a [0, 2*π*] range.
(AVI)

S2 Video Odd crystal formation with no diversity. Numerical simulations starting from random narrowly distributed initial condition for a population of *N* = 33 identical oscillators, *σ* = 0 with different polarity.
(AVI)

S3 Video Adaptability to small diversity. The movie shows crystal growth as in S2, but with small diversity. Particles exchange takes place in order to adapt the pattern to diversity. *N* = 21, *σ* = 0.25.
(AVI)

S4 Video Robustness to perturbation. One element is perturbed after pattern formation. The structure quickly reacts and accomodate the perturbed element in a new similar pattern. No diversity is present in this simulation, though this feature persists in presence of small diversity. *N* = 21.
(AVI)

S5 Video Diversity induced collective pulsations. Diversity makes the structure vibrate; under certain conditions, the structure remains oscillating in a pseudo-regular fashion, as in the present movie. *N* = 17, *σ* = 0.4.
(AVI)

S6 Video Meiosis. Increasing diversity the pattern splits and the resulting subforms relate each other as independent entities. *N* = 15, *σ* = 0.5.
(AVI)

S7 Video Dynamic pattern competition and disorder. Further increasing diversity the dynamic activity grows and pattern lifetimes decrease. *N* = 29, *σ* = 0.7.
(AVI)

## References

[1] Kauffman SA. The Origins of Order: Self Organization and Selection in Evolution. Oxford University press; 1993.

[2] Jantsch E. The self-organizing universe. Oxford: Pergamon press; 1980.

[3] Percec V, Glodde M, Bera TK, Miura Y, Shiyanovskaya I, Singer KD, Balagurusamy V, Heiney SK, Schnell PA, Rapp I, Spiess A, Hudson HW, Duan SD. Self-organization of supramolecular helical dendrimers into complex electronic materials. Nature. 2002; 417: 384–387. doi: 10.1038/nature01072 10.1038/nature01072 12352988

[4] Naka K, Carney CK. Biomineralization I: Crystallization and Self-Organization Process. Berlin: Springer-Verlag; 2007.

[5] Tadros TF. Self-Organized Surfactant Structures. London:Wiley and sons; 2011.

[6] Axel HE, Müller OB. Self Organized Nanostructures of Amphiphilic Block Copolymers I. Berlin: Springer Science and Business Media; 2011.

[7] Gerstman BS, Chapagain PP. Self-organization in protein folding and the hydrophobic interaction. J. Chem. Phys. 2005; 123: 054901 doi: 10.1063/1.1990110 16108687 10.1063/1.1990110

[8] Sweetman AM, Jarvis SP, Sang H, Lekkas I, Rahe P, Wang Y, Wang J, Champness NR, Kantorovich L, Moriarty P. Mapping the force field of a hydrogen-bonded assembly. Nature Communications. 2014; 5: 3921 doi: 10.1038/ncomms4931 24875276 10.1038/ncomms4931 PMC4050271

[9] Mao C, Sun W, Seeman NC. Assembly of Borromean rings from DNA. Nature. 1997; 386 (6621): 137–138. doi: 10.1038/386137b0 9062186 10.1038/386137b0

[10] Rosen BM, Wilson CJ, Wilson DA, Peterca M, Mohammad R, Imam A and Percec V. Dendron-Mediated Self-Assembly, Disassembly, and Self-Organization of Complex Systems. Chem. Rev. 2009; 109 (11): 6275–6540. doi: 10.1021/cr900157q 19877614 10.1021/cr900157q

[11] Millor J, Pham-Delegue M, Deneubourg JL, and Camazine S. Self-organized defensive behavior in honeybees. Proc Natl Acad Sci U. S A. 1999; 96(22): 12611–12615. doi: 10.1073/pnas.96.22.12611 10535970 10.1073/pnas.96.22.12611 PMC23012

[12] Athanasios K.A., Eswaramoorthy R., Hadid P., and Hu J.C. Self-Organization and the Self-Assembling Process in Tissue Engineering. Annual Review of Biomedical Engineering. 2013; 15: 115–136. doi: 10.1146/annurev-bioeng-071812-152423 10.1146/annurev-bioeng-071812-152423 PMC4420200 23701238

[13] Bonabeau E, Dorigo M, Theraulaz G. Swarm intelligence From natural to artificial systems. Oxford University Press; 1999.

[14] Winfree AT. The Prehistory of the Belousov-Zhabotinsky Oscillator. Journal of Chemical Education. 1984; 61: 661–663. doi: 10.1021/ed061p661

[15] Orlik M. Self-Organization in Electrochemical Systems I. Berlin: Springer Science and Business Media; 2012.

[16] Winfree A.T. Biological rhythms and the behavior of populations of coupled oscillators. Journal of Theoretical Biology. 1967; 16: 15–42. doi: 10.1016/0022-5193(67)90051-3 6035757 10.1016/0022-5193(67)90051-3

[17] Ma J., and Tang J. A review for diversity in neuron and neuronal network. Nonlinear Dynamics. 2017; 89: 1569–1578.

[18] Ma J., Quin H., Song X., and Chu R. Pattern selection in neuronal network driven by electric autapses with diversity in time delays. Science China Technological Sciences. 2015; 58: 2038–2045.

[19] Ma J., and Tang J. A review for dynamics of collective behaviours of network of neurons. Science China Technological Sciences. 2015; 58: 2038–2045. doi: 10.1007/s11431-015-5961-6

[20] Wang C., He Y., Ma J., Huang L. Parameters Estimation, Mixed Synchronization, and Antisynchronization in Chaotic Systems. Complexity. 2014; 20: 64–73. doi: 10.1002/cplx.21497

[21] Chen J.-X., Zhang H., Qiao l.-Y., Liang H., and Sun W.-G. Interaction of excitable waves emitted from two defects by pulsed electric fields. Communications in Nonlinear Science and Numerical Simulations. 2018; 54: 202–209. doi: 10.1016/j.cnsns.2017.05.034

[22] Lv M., and Ma J. Multiple modes of electrical activities in a new neuron model under electromagnetic radiation. Neurocomputing. 2016; 205: 375–381. doi: 10.1016/j.neucom.2016.05.004

[23] Chen J.-X., Peng L., Ma J., and Ying H.P. Liberation of a pinned spiral wave by a rotating electric pulse. EPL. 2014; 107: 38001 doi: 10.1209/0295-5075/107/38001

[24] Chen J.-X., Guo M.M., and Ma J. Termination of pinned spirals by local stimuli. EPL. 2016; 113: 38004 doi: 10.1209/0295-5075/113/38004

[25] Ansari H. A., and Smolin L. Self-organized criticality in quantum gravity. Classical and Quantum Gravity. 2008; 25: 095016 doi: 10.1088/0264-9381/25/9/095016

[26] Fatima B., and Shah A. Self organization based energy management techniques in mobile complex networks: a review. Complex Adaptive Systems Modeling. 2015; 3: 2 doi: 10.1186/s40294-015-0008-1

[27] Ulrich H, Probst G, Self-Organization and Management of Social Systems Berlin: Springer Science and Business Media; 2012.

[28] Horsthemke W, Lefever R. Noise-induced Transitions. Berlin: Springer-Verlag; 1984.

[29] San Miguel M. Toral R., Stochastic effects in physical systems In: Instabilities and nonequilibrium structures VI (eds. Tirapegui J. M. E. and Tiemann R.). Dordrecht: Kluwer academic publishers; 2000 pp. 35–120.

[30] Tessone C, Zanette D. Toral R., Global firing induced by network disorder in ensembles of active rotators. Eur. Phys. J. B. 2008; 62: 319–326. doi: 10.1140/epjb/e2008-00162-5

[31] Chen H, and Zhang J. Diversity-induced coherence resonance in spatially extended chaotic systems. Physical Review. 2008; E 77: 026207. 10.1103/PhysRevE.77.026207 18352103

[32] Tessone CJ, Scirè A, Toral R, Colet P. Theory of collective firing induced by noise or diversity in excitable media. Physical Review. 2007; E 75: 016203. 10.1103/PhysRevE.75.016203 17358231

[33] Atmanspacher H, Bishop R. Between Chance and Choice: Interdisciplinary Perspectives on Determinism. Exeter: Imprint Academic; 2007.

[34] Hirschberg J, Brunsveld L, Ramzi A, Vekemans J, Sijbesma R., Meijer EW. Helical self-assembled polymers from cooperative stacking of hydrogen-bonded pairs. Nature. 2000; 407: 167–170. doi: 10.1038/35025027 11001050 10.1038/35025027

[35] Riger S, Lavrakas PJ. Community ties: Patterns of attachment and social interaction in urban neighborhoods. American Journal of Community Psychology. 1981; 9(1): 55–66. doi: 10.1007/BF00896360

[36] Kuramoto Y. H. Araki, ed. Lecture Notes in Physics, International Symposium on Mathematical Problems in Theoretical Physics 39. New York: Springer-Verlag; 1975; 420.

[37] Kuramoto Y. Chemical Oscillations, Waves, and Turbulence. New York: Springer-Verlag; 1984.

[38] Acebrón JA, Bonilla LL, Vicente P, Conrad J, Ritort F, Spigler R. The Kuramoto model: A simple paradigm for synchronization phenomena. Reviews of Modern Physics. 2005; 77: 137–185. doi: 10.1103/RevModPhys.77.137

[39] Adler R. A Study of Locking Phenomena in Oscillators. Proceedings of the IRE. 1946; 34(6): 351–357. doi: 10.1109/JRPROC.1946.229930

[40] Brush GB. History of the Lenz-Ising Model. Reviews of Modern Physics. 1967; 39: 883–893. doi: 10.1103/RevModPhys.39.883

[41] Lipowski A., Ferreira L.F., Lipowska D., Gontarek K. Phase transitions in Ising models on directed networks. Physical Review E. 2015; 92: 052811 doi: 10.1103/PhysRevE.92.052811 10.1103/PhysRevE.92.052811 26651748

